# Association between calf circumference and incontinence in Chinese elderly

**DOI:** 10.1186/s12889-023-15324-4

**Published:** 2023-03-11

**Authors:** Lin Li, Feilong Chen, Xiaoyan Li, Yiyuan Gao, Silin Zhu, Xiyezi Diao, Ning Wang, Tao Xu

**Affiliations:** 1 Clinical Medical Center, National Research Institute for Family Planning, Beijing, China; 2 Department of Epidemiology and Biostatistics Institute of Basic Medical Sciences, Chinese Academy of Medical Sciences & School of Basic Medicine, Peking Union Medical College, Beijing, China; 3 Center for Health Quality, National Research Institute for Family Planning, Beijing, China; 4 Department of Statistics, Central China Normal University, Hubei, China

**Keywords:** The elderly, Calf circumference, Incontinence, Sarcopenia, Risk factor

## Abstract

**Background:**

The objective of this study was to analyze the association between calf circumference and incontinence in Chinese elderly, and to find out the maximal cut-off point by gender for the use of calf circumference in screening for incontinence.

**Methods:**

In this study, participants were from the 2018 Chinese Longitudinal Healthy Longevity Survey (CLHLS). The maximal calf circumference cut-off point and other incontinence-related risk factors were explored using receiver operating characteristic (ROC) curves and logistic regression analysis.

**Results:**

The study included 14,989 elderly people (6,516 males and 8,473 females) over 60. The prevalence of incontinence in elderly males was 5.23% (341/6,516), significantly lower than females, which was 8.31% (704/8,473) (*p* < 0.001). There was no correlation between calf circumference < 34 cm in males and < 33 cm in females and incontinence after adjusting the confounders. We further stratified by gender to predict incontinence in elderly based on the Youden index of ROC curves. We found the association between calf circumference and incontinence was the strongest when the cut-off points were < 28.5 cm for males and < 26.5 cm for females, with an odds rate (OR) value of 1.620 (male, 95%CI: 1.197–2.288) and 1.292 (female, 95%CI: 1.044–1.600) after adjusting the covariates, respectively.

**Conclusions:**

Our study suggests that calf circumference < 28.5 cm in males and < 26.5 cm in females is a risk factor for incontinence in the Chinese elderly population. Calf circumference should be measured in routine physical examination, and timely interventions should be made to reduce the risk of incontinence in subjects with calf circumference less than the threshold.

**Supplementary Information:**

The online version contains supplementary material available at 10.1186/s12889-023-15324-4.

## Background

Population aging has become an important issue in China. According to the Seventh National Population Census of the National Bureau of Statistics, by the end of 2020, the elderly population aged 60 and above in China has reached 264 million, accounting for 18.7% of the total population [1], thus China has the largest elderly population in the world. With the increased aging population in China, age-related diseases have attracted more and more attention. Is there a link between sarcopenia and incontinence, given that they are both associated with aging? So far, only a few international studies have attempted to reveal the relationship between sarcopenia and incontinence, but no firm conclusions have been reached. Studies have shown that sarcopenia involved the weakness of abdominal muscles and pelvic floor muscles, leading to urinary incontinence(UI) as the urethra could not generate sufficient pressure to resist increases in intravesical pressure. Therefore, sarcopenia was considered to increase the susceptibility to UI in elderly people [2].

Sarcopenia is defined as age-related loss of muscle mass, decreased muscle strength, and/or lower physical performance [3]. The Global Leadership Initiative on Malnutrition (GLIM) approaches for diagnosing malnutrition included technical approaches(such as bioelectrical impedance analysis, dual-energy x-ray absorptiometry, computerized tomography and Ultrasound) and clinical approaches(such as calf circumference, mid-arm circumference and physical examination), and recommended that "cut-off values were calf circumference in male < 33 cm and in female < 32 cm" [4]. Asian Working Group for Sarcopenia (AWGS): 2019 Consensus update on sarcopenia diagnosis and treatment pointed out that "calf circumference, as a simple method to assess skeletal muscle mass in limbs, can be used for effective screening of sarcopenia," and the diagnostic parameters were "calf circumference in male < 34 cm and in female < 33 cm" [5]. Calf circumference is an anthropometric variable correlated with skeletal muscle mass (ASM), and estimates cross-sectional muscle area, subcutaneous fat and skin fold. When elderly people have very little subcutaneous fat, calf circumference is more representative of muscle mass [6].

The problem of incontinence among the elderly population has simultaneously become increasingly prominent. The negative impact of incontinence on the quality of life and social dignity of elders has become a serious medical and social problem due to the significant improvement in living standards. Incontinence mainly includes urinary incontinence and fecal incontinence. The International Continence Society defined that urinary incontinence (UI) is the complaint of any involuntary leakage of urine, which brings inconvenience to patients in social activities and personal hygiene [7]. Fecal incontinence (FI) is defined as the involuntary loss or passage of solid or liquid stools [8]. Studies on the epidemiology of incontinence have found that the incidence of UI in males is 5.4% [9], and 25% among Norwegian females [10]. The incidence of FI among older Brazilian males was 4.7% and 7.3% among females [11]. A survey from Peking Union Medical College Hospital in China showed that the prevalence of UI and FI in adult females was 30.9% and 0.43%, and increased with age [12, 13].

In this study, calf circumference was used as a screening index to analyze the association between calf circumference and incontinence, aiming to provide a simpler, more convenient and effective indicator for clinical prevention and diagnosis of incontinence in the elderly population.

## Methods

### Participants

The information was obtained from the Chinese Longitudinal Health and Longevity Survey (CLHLS), a nationwide, prospective cohort study of middle-aged and elderly people who were community-dwelling and institutionalized in China, initiated by the Center for Healthy Aging and Family Studies at Peking University. It covered most of China's provinces, and aimed to understand the health status of China's elderly, and related social, behavioral, and biological factors. The study conducted the first baseline survey in 1998, followed by a follow-up every two to three years, and covered the eastern, central, and western regions of China. Details such as sampling design, and data quality assessment can be found in previous studies [14, 15].

**Fig. 1:**
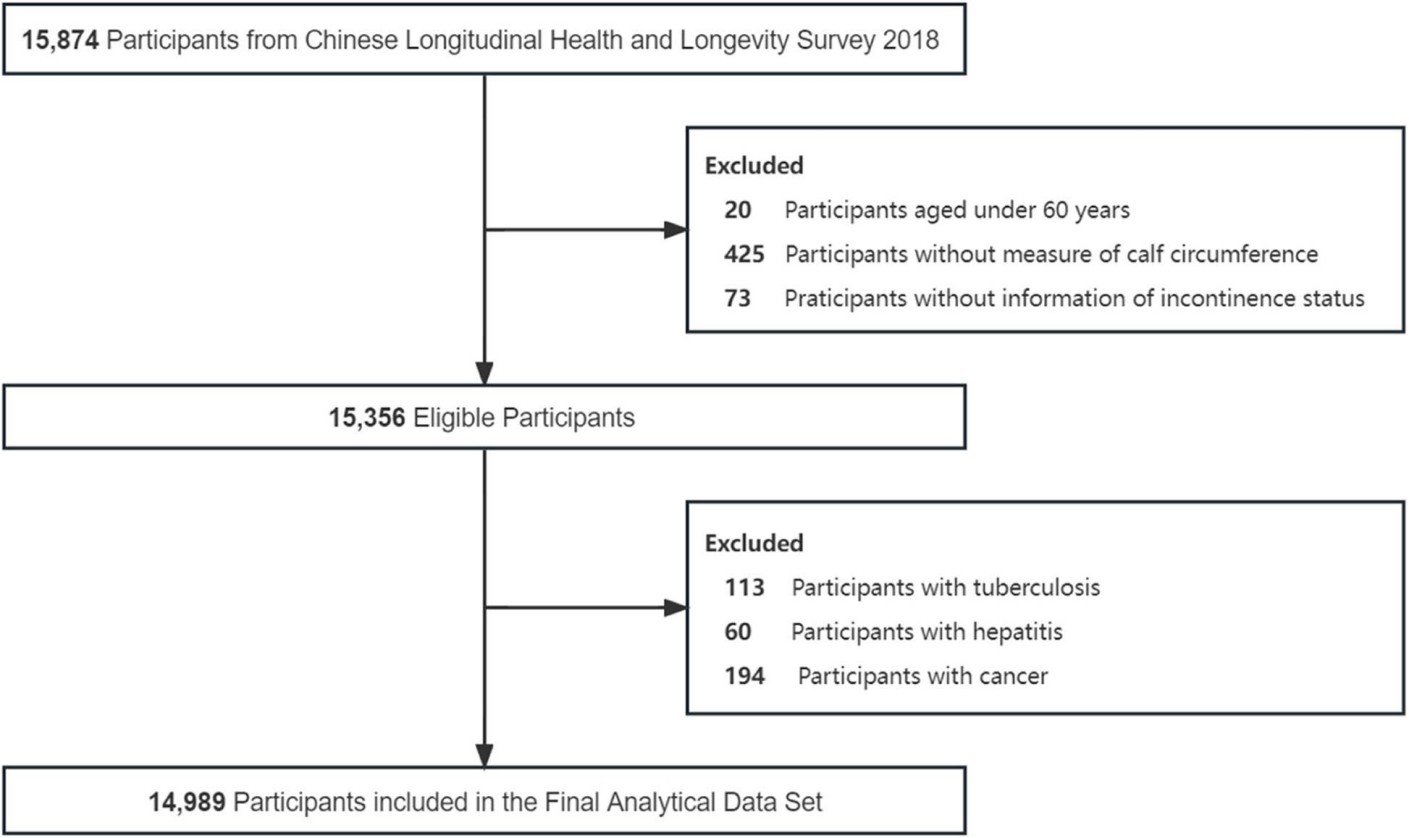
Flowchart of Participants selection

Our study used data obtained from the Seventh CLHLS cross-sectional survey conducted in 2018. The survey collected health data of 15,874 elderly people from 23 provincial administrative regions in China. We excluded individuals with missing data on calf circumference and incontinence status, as well as individuals under the age of 60, and filtered out the abnormal values of calf circumference. We further excluded individuals with chronic wasting diseases including tuberculosis, hepatitis and cancer. In the end, we included 14,989 subjects, of whom 43.47% were male and 56.53% were female (Fig. 1).

### Data collection

This study collected the information on main research indicators (calf circumference length, incontinence status), demographic characteristics (gender, socioeconomic status, waist circumference, body mass index [BMI], education level, history of pregnant [only in females]), lifestyle characteristics (smoking status, drinking status, physical exercise, limited ability of daily activities, intake of fruits and vegetables) and history of diseases (including history of falling, history of respiratory diseases, urinary diseases, stroke or cardiovascular diseases, and nerves system disease). Calf circumference, waist circumference, height, and weight were measured by highly trained doctors and nurses. Data on demographic information, lifestyle characteristics, incontinence status, and history of diseases were obtained through face-to-face interviews using internationally compatible questionnaires, which had demonstrated good reliability and validity. All investigators received strict training in advance.

The study was approved by the Biomedical Ethics Committee of Peking University (IRB00001052-24,713,074). All participants signed a written informed consent form.

### Definition of incontinence status

In this study, the status of incontinence was assessed through three options in the questionnaire. These included "able to control defecation," "occasional or sometimes incontinence," and "using catheters to assist in controlling incontinence or unable to control." "Able to control defecation" was defined as non-incontinence, and the latter two options were defined as the existence of incontinence.

### Measurement of calf circumference

The calf circumferences were measured by trained investigators. The subjects were in sitting position, bending their knees and hips 90 degrees, with feet placed naturally on the ground. Facing the subjects, the investigator placed an inelastic belt ruler around the calf, did not compress the subcutaneous tissue, and moved along the length of the calf to obtain the maximum perimeter. Each leg was measured twice, and the average value was taken as the circumference value of each leg. The measured value of the calf circumference of the two legs was then averaged and recorded as the final measured value of the calf circumference [16]. The measurement of calf circumference was in cm, accurate to one decimal place [17]. All the staffs received strict train before they were allowed to performed the measurement, and they were required to strictly follow the standard operating procedures (SOPs). Besides, participants also received pre-education to ensure that they would actively cooperate with the researchers. Other detailed quality control measures could be seen on CLHLS website [18].

Based on the 2019 Consensus released by the AWGS, the cut-off points of sarcopenia were indicated by the use of calf circumference < 34 cm for males and < 33 cm for females in different places such as communities and nursing homes [19]. Therefore, our study used these cut-off values to divide the calf circumference. Small calf circumference was defined as < 34 cm for males and < 33 cm for females, and the opposite was defined as thick calf circumference.

### Covariates

The covariates included demographic characteristics (gender, socioeconomic status, waist circumference, BMI, education, history of childbirth) and lifestyle characteristics (smoking history, drinking history, participation in exercise, limitation of daily activities, intake of fruits and vegetables, and history of respiratory diseases).

According to the Dietary Guidelines for Chinese Residents 2022 [20], the appropriate BMI level for Chinese people over 65 was 20.0 kg/m2–26.9 kg/m2, and BMI > 27 kg/m2 was associated with overweight or obesity [21]. The definition of socioeconomic status was based on subjects' subjective life feelings: those who thought that their living conditions were "very rich" or "relatively rich" were defined as "better," "average" was defined as "average," and "more difficult" and "very difficult" were defined as "poor." Waist circumference was measured using an inelastic tape at the end of quiet breathing [22]. Education levels were judged by the length of school time reported by the subjects. Those who had never attended school were defined as "illiterate," those who attended school for 1–10 years were defined as "primary or junior high school," and those who attended school for more than 10 years were defined as "university or above." Females who had never pregnant defined as "No history of pregnant," those who had pregnant once were defined as "Having a history of pregnant." Lifestyle characteristics covariates were classified according to the subjects' answers to the questionnaires. History of respiratory disease was defined as the subject having suffered from chronic respiratory disease such as bronchitis, emphysema, asthma, or pneumonia formerly or presently; Urological disease history was defined as the subject having chronic nephritis, prostatitis, etc., and history of neurological disease was defined as participants who had Parkinson's disease, dementia or epilepsy.

### Statistical analysis

SAS version 9.4 and R 4.2.2 software were used for data analysis. Two-tailed tests were conducted and *p* < 0.05 was defined as statistically significant. For baseline information, different groups were divided according to gender and incontinence status. Continuous normal distribution data were described by mean $\pm$ standard deviations, and Student’s t-tests were used for comparison between groups. The classified data were described by numbers and percentages, and were compared using the Chi-squared test.

To clearly illustrated the association between calf circumference and incontinence, we modeled calf circumference against incontinence and used a restricted cubic spline with three knots located at the 25th, 50th, and 75th percentiles to flexibly model the underlying relationship. Two different methods were used to divide the calf circumferences. Firstly, according to AWGS’s 2019 Consensus, male calf circumference < 34 cm and female < 33 cm were defined as small calf circumference, while male ≥ 34 cm and female ≥ 33 cm were defined as thick leg circumference [19]. Secondly, we divided the calf circumference based on the maximal calf circumference cut-off points by calculating the greatest Youden index through the ROC curves. During analysis, we stratified the data by gender due to the gender differences in normal range of calf circumference and prevalence of incontinence among elderly people. The results showed that the Youden index was the greatest when 28.5 cm (male, Supplementary Table 1) and 26.5 cm (female, Supplementary Table 2) were used as cut-off points. Therefore, we defined small calf circumference as < 28.5 cm for males, and < 26.5 cm for females, and thick calf circumference was defined as calf circumference ≥ 28.5 cm for males and ≥ 26.5 cm for females.

We used logistic regression models to assess the associations between calf circumference and incontinence. We included the calf circumference as a continuous variable, a binary variable (male < 34 cm, female < 33 cm), and a binary variable (male < 28.5 cm, female < 26.5 cm) in the model to compare the relationship between different divisions of calf circumference and incontinence. Furthermore, we fit two models stratified by gender. Model 1 did the univariate analysis. Model 2 further adjusted age, history of falling, smoking status, drinking status, physical exercise, limited ability of daily activities, intake of vegetables and fruits, socioeconomic status, waistline circumference, BMI, education, history of respiratory diseases, history of urinary system diseases, history of stroke or cardiovascular diseases, history of nerves system disease and history of pregnant (only in females). We calculated the odds rates (ORs) and 95% confidence intervals (95% CI) to estimate the association between calf circumference and incontinence among males and females.

## Results

### Characteristics of participants

Our study included 14,989 participants aged above 60. Among them, 6,516 were males (43.47%) and 8,473 were females (56.53%). According to Table 1, for both genders, all demographic characteristics were statistically different between different incontinence status groups, except for history of pregnant. In male populations, there were statistical differences in drinking and smoking status between groups, while there were no such differences among females. For both males and females, taking nutritional supplements was not associated with incontinence.

**Table 1:** Baseline information of participants

Variables	Male (*n* = 6516)		*p*	Female (*n* = 8473)		*p*
	Not Incontinence	Incontinence		Not Incontinence	Incontinence	
**Total**	*n* = 6175	*n* = 341	< 0.001	*n* = 7769	*n* = 704	< 0.001
Age in years, mean (sd)	82.69 (10.70)	93.43 (8.85)	< 0.001	86.20 (11.90)	97.06 (7.49)	< 0.001
Calf circumference^1^, cm	32.72 (5.84)	30.14 (6.81)	< 0.001	29.88 (6.11)	26.64 (6.82)	< 0.001
Calf circumference^2^, %			< 0.001			< 0.001
≥ 34 (Male) /33 (Female) < 34 (Male) /33 (Female)	2601 (42.11)	90 (26.39)		2196 (28.27)	89 (12.64)	
	3574 (57.89	251 (73.61)		5573 (71.73)	615 (87.36)	
Calf circumference^3^, %			< 0.001			< 0.001
≥ 28.5 (Male) /26.5 (Female)	5080 (82.27)	206 (60.41)		5754 (74.06)	320 (45.45)	
< 28.5 (Male) /26.5 (Female)	1095 (17.73)	135 (39.59)		2015 (25.94)	384 (54.55)	
Age group, %			< 0.001			< 0.001
60–70	770 (12.47)	4 (1.17)		772 (9.94)	2 (0.28)	
71–80	1787 (28.94)	28 (8.21)		1765 (22.72)	24 (3.41)	
81–90	1737 (28.13)	62 (18.18)		1910 (24.58)	79 (11.22)	
91–100	1351 (21.88)	129 (37.83)		1668 (21.47)	199 (28.27)	
> 100	530 (8.58)	118 (34.60)		1654 (21.29)	400 (56.82)	
Waist-to-Hip Ratio (WHR), %			0.033			< 0.001
< 0.9 (Male) /0.8 (Female)	2729 (44.19)	171 (50.15)		2792 (35.94)	356 (50.57)	
≥ 0.9 (Male) /0.8 (Female)	3446 (55.81)	170 (49.85)		4977 (64.06)	348 (49.43)	
Socioeconomic status, %			< 0.001			< 0.001
Poor	613 (10.02)	55 (16.42)		788 (10.25)	139 (20.26)	
General	4144 (67.71)	224 (66.87)		5555 (72.26)	471 (68.66)	
Good	1363 (22.27)	56 (16.72)		1345 (17.49)	76 (11.08)	
Education level, %			< 0.001			< 0.001
Illiterate	1363 (26.45)	132 (41.90)		4489 (66.23)	505 (80.03)	
Primary & Middle school	3030 (58.79)	137 (43.49)		1950 (28.77)	95 (15.06)	
University and above	761 (14.77)	46 (14.60)		339 (5.00)	31 (4.91)	
BMI, %			< 0.001			< 0.001
Emaciation	1616 (26.17)	184 (53.96)		2805 (36.11)	472 (67.05)	
Normal	3800 (61.54)	123 (36.07)		3961 (50.98)	173 (24.57)	
Overweight or obesity	759 (12.29)	34 (9.97)		1003 (12.91)	59 (8.38)	
Pregnant history, %			/			0.509
Never	/	/		115 (1.70)	13 (2.06)	
Equal or more than once	/	/		6654 (98.30)	619 (97.94)	
Frequently eat fruits & vegetables, %			< 0.001			< 0.001
No	120 (1.95)	29 (8.53)		192 (2.48)	68 (9.69)	
Yes	6033 (98.05)	311 (91.47)		7556 (97.52)	634 (90.31)	
Smoking status, %			< 0.001			0.291
No	2591 (42.56)	184 (54.44)		7000 (91.94)	631 (90.79)	
Yes	3497 (57.44)	154 (45.56)		614 (8.06)	64 (9.21)	
Drinking status, %			< 0.001			0.579
No	3313 (54.59)	221 (65.77)		6765 (89.24)	615 (89.39)	
Yes	2756 (45.41)	115 (34.23)		816 (10.76)	73 (10.61)	
Exercise, %			< 0.001			< 0.001
No	3356 (55.21)	234 (70.27)		5022 (65.97)	548 (79.65)	
Yes	2723 (44.79)	99 (29.73)		2590 (34.03)	140 (20.35)	
Limited daily activities, %			< 0.001			< 0.001
No	4479 (72.72)	52 (15.29)		4866 (62.84)	72 (10.26)	
Yes	1680 (27.28)	288 (84.71)		2878 (37.16)	630 (89.74)	
Take dietary supplements, %			0.341			0.849
No	5482 (90.03)	298 (88.43)		6738 (87.96)	606 (88.21)	
Yes	607 (9.97)	39 (11.57)		922 (12.04)	81 (11.79)	
History of Fall, %			< 0.001			
No	4981 (81.94)	217 (64.97)		5784 (75.77)	452 (66.47)	
Yes	1098 (18.06)	117 (35.03)		1850 (24.23)	228 (33.53)	
Respiratory diseases history, %			< 0.001			< 0.001
No	5447 (88.21)	276 (80.94)		7196 (92.62)	629 (89.35)	
Yes	728 (11.79)	65 (19.06)		573 (7.38)	75 (10.65)	
Urinary disease history, %			< 0.001			0.258
No	5612 (90.88)	281 (82.40)		7691 (99.00)	700 (99.43)	
Yes	563 (9.12)	60 (17.60)		78 (1.00)	4 (0.57)	
Stroke or cardiovascular disease history, %			< 0.001			< 0.001
No	5520 (89.39)	236 (69.21)		7112 (91.54)	573 (81.39)	
Yes	655 (10.61)	105 (30.79		657 (8.46)	131 (18.61)	
Nervous system disease history, %			< 0.001			< 0.001
No	6075 (98.38)	285 (83.58)		7600 (97.82)	573 (81.39)	
Yes	100 (1.62)	56 (16.42)		169 (2.18)	131 (18.61)	

Continuous normal distribution data were described by mean $($standard deviations), and Student’s t-tests were used for comparison between groups. The categorized data were described by numbers (%), and were compared using the Chi-squared test

Calf circumference^1^: Take calf circumference as a continuous variable. Calf circumference^2^: The calf circumference is divided into two categories. The cut-off value is 34 cm for males, and 33 cm for females. Calf circumference^3^: The calf circumference is divided into two categories. The cut-off value is 28.5 cm for males, and 26.5 cm for females

Additionally, for the two different methods of division of calf circumference, there were both statistical differences in the distribution of different incontinence groups in both genders. When calf circumference was classified using a cut-off value of 34 cm for males and 33 cm for females, the prevalence of incontinence was 6.56% for male with thick calf circumference (≥ 34 cm) and 3.35% for those with small calf circumference; for female subjects with thick calf circumference (≥ 33 cm), the prevalence was 9.94%, and the prevalence was 3.89% for those with thin. The prevalence of incontinence significantly increased when using 28.5/26.5 cm as cut-off points for male and female population. 10.98% of males with small calf circumference (< 28.5 cm) suffered from incontinence, while this proportion was 16.01% among females with small calf circumference (< 26.5 cm).

### Association of calf circumference groups with incontinence status among elderly people

**Fig. 2:**
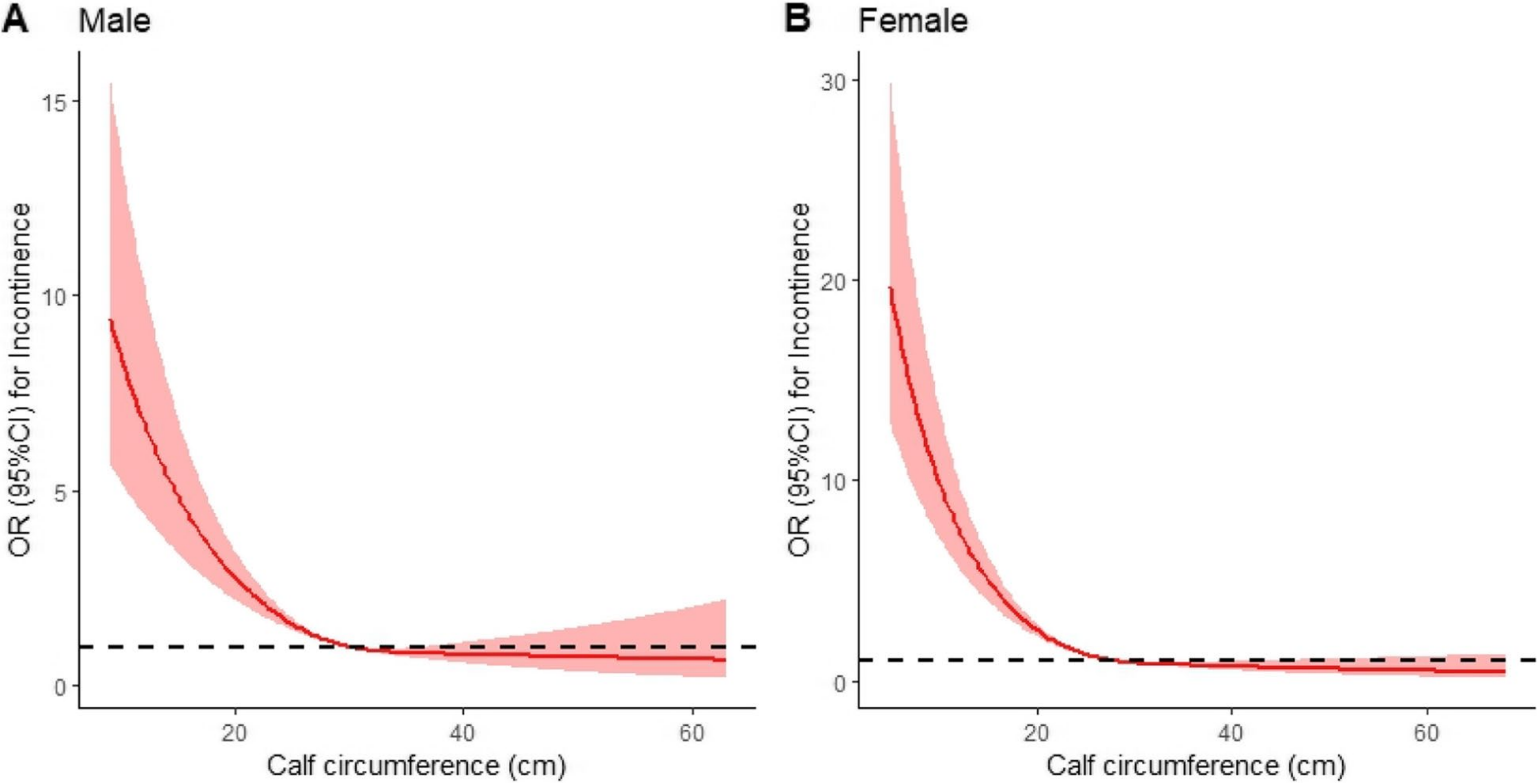
Association of calf circumference and incontinence by sex. **A** Calf circumference restricted cubic spline regression with 3 knots in male participants; red solid line represents association between calf circumference and incontinence, shaded areas are 95% CIs. p for non-linear < 0.001. **B** Calf circumference restricted cubic spline regression with 3 knots in female participants; red solid line represents association between calf circumference and incontinence, shaded areas are 95% CIs. *p* for non-linear < 0.001

The prevalence of incontinence was 8.31% in elderly females, significantly higher than elderly males (5.23%) (*p* < 0.001) (Table 1). We found a significant non-linear association between calf circumference and risk of incontinence both in males and females (Fig. 2). To the left of the inflection point, smaller calf circumference was a risk factor for incontinence and the risk of incontinence decreased significantly with increasing calf circumference, whereas to the right of the inflection point, increasing calf circumference was a protective factor for incontinence, but increasing calf circumference had little effect on reducing the risk of incontinence.

Table 2 shows the results of the logistic regression. The results show that the association strength between calf circumference and incontinence was the highest when the cut-off points of calf circumference were 28.5 cm for males and 26.5 cm for females. The ORs were 3.040 for males (95% CI: 2.420, 3.809) and 3.427 for females (95% CI: 2.930, 4.010), indicating that the risk of incontinence in males and females with smaller calf circumference was 3.040 and 3.427 times higher than those with thicker calf circumference, respectively. After adjusting for the covariates, the strength of the association became smaller, but still remained statistically significant. Compared with thick calf circumference, subjects with thin calf circumference had a 62.0% (male, OR: 1.620, 95% CI: 1.197, 2.188) and 29.2% (female, OR: 1.292, 95% CI: 1.044,1.600) increased risk of developing incontinence, respectively. A significant association between calf circumference and incontinence was found in Model 1 when the calf circumference entered the models in the forms of binary variables (cut-off points 34 cm for males and 33 cm for females), but disappeared in Model 2 after adjusting the confounders.

**Table 2:** Association of calf circumference and incontinence by gender

	Male		Female	
	Not Incontinence	Incontinence	Not Incontinence	Incontinence
**Continuous calf circumference **^**1**^				
Model 1	1(reference)	0.927(0.910,0.944)*	1(reference)	0.909(0.897,0.922)*
Model 2	1(reference)	0.972(0.948,0.996)*	1(reference)	0.985(0.967,1.004)
**Binomial calf circumference (Male < 34 cm and Female < 33 cm as cut-off points)**				
Model 1	1(reference)	2.029(1.593,2.607)*	1(reference)	2.722(2.181,3.440)*
Model 2	1(reference)	1.218(0.882,1.691)	1(reference)	1.118(0.820,1.540)
B**inomial calf circumference (Male < 28.5 cm and Female < 26.5 cm as cut-off points)**				
Model 1	1(reference)	3.040(2.420,3.809)*	1(reference)	3.427(2.930,4.010)*
Model 2	1(reference)	1.620(1.197,2.188)*	1(reference)	1.292(1.044,1.600)*

Model 1 was univariate model; Model 2 further adjusted age, history of falling, smoking status, drinking status, physical exercise, limited ability of daily activities, intake of vegetables and fruits, socioeconomic status, waistline circumference, BMI, education, history of respiratory diseases, history of urinary system diseases, history of stroke or cardiovascular diseases, history of nerves system disease and history of pregnant (only in females)

^1^The calf circumference was included in the model as a continuous variable

^2^The calf circumference was classified with male < 34 cm and female < 33 cm as cut-off points

^3^The calf circumference was classified with male < 28.5 cm and female < 26.5 cm as cut-off points

^*^ *p* < 0.05

Supplementary tables 3, 4, and 5 show the detailed parameters of every variable in the models. With the increase of age, the association strength also increased, and these trends appeared in both men and women, indicating that age is an important risk factor for incontinence. However, the effect of BMI was opposite. The risk of incontinence in people with normal and overweight or obese BMI was significantly lower than thinner people.

## Discussion

This study is the first to examine the association between calf circumference and incontinence in elderly individuals living in Chinese communities. The results indicated that calf circumference was statistically associated with incontinence in the elderly. Calf circumferences of less than 28.5 cm in males and less than 26.5 cm in females were identified as risk factors for incontinence, which were 1.620 (95%CI: 1.197, 2.188) and 1.292 (95%CI: 1.044, 1.600) times greater risk of incontinence than those with larger calf circumference.

A total of 14,989 elderly people over 60 years old were included in this study, with males accounting for 43.47% and females 56.53%. The research showed that the prevalence of incontinence in elderly males was 5.23%, significantly lower than among elderly females (8.31%). Existing studies mostly classify incontinence into UI and FI. For example, the prevalence of FI in female over 65 years old in Taiwan, China was 9.3% [23]. The prevalence of liquid FI in older US females was 7.9% and that of solid FI was 6.5% [24]. The results of The EPIC Study, conducted jointly by Canada and five other countries, showed the prevalence of UI in elderly males over 60 years old was 4.14% and that in elderly females was 13.23% [25]. The results of the above study are slightly different from those of this study, which may stem from the different types of incontinence studied.

The GLIM pointed out that the recommended cut-off values in the diagnosis of malnutrition were calf circumference in male < 33 cm and in female < 32 cm [4]. They were lower than the diagnostic parameters from the Chinese expert consensus on diagnosis and treatment for elderly with sarcopenia (2021), which for sarcopenia were "calf circumference < 34 cm for men and < 33 cm for women"[3]. The GLIM was aimed at the entire adult population including the young, middle-aged and the elderly population, while the Chinese expert consensus was aimed at the elderly population only. Therefore, calf circumference of 34 cm in males and 33 cm in females were used as the grouping index for this study. After adjusting for confounders, we found that calf circumference < 34 cm in males and < 33 cm in females were not statistically associated with incontinence in elderly. So, are there any other cutoff values for calf circumference associated with incontinence in elderly people? No studies have been reported. Based on the GLIM's advice, receiver operating characteristic(ROC) curve could be used to identify a new maximal cutoff when there were no data from a reference population[4].Further stratification by gender to predict incontinence in elderly based on the sensitivity, specificity, and the Youden index of ROC curve, we found that the association strength between calf circumference and incontinence was the highest when the cut-off points of calf circumference were < 28.5 cm for males and < 26.5 cm for females, and the risk of incontinence in males with calf circumference < 28.5 cm and females with calf circumference < 26.5 cm was 3.040 times (males) and 3.427 times (females) higher than people with thicker calf circumference. The findings of NHANES suggested that, for elder people, a moderately low calf circumference (1 SD below the mean) can be adequate for sarcopenia diagnosis / screening and all calf circumference mean values differed among the ethnic and race groups [26]. Meanwhile, studies had shown that UI was closely related to musculoskeletal conditions and impaired function among elderly people, and it was suggested that markers of sarcopenia could be used as important clinical predictors of UI in elderly females [27]. Therefore, we speculate that calf circumference can be used as a predictor of incontinence in the elderly people. Recent studies in Japan also found that the average calf circumference of patients with FI was significantly lower than that of patients without FI, which was statistically significant and consistent with our findings [28].

Previous studies had found that pelvic muscle mass decreased along with general muscle mass in elderly patients with UI, and atrophy and weakness of pelvic muscle led to UI [29]. Another study found a significant increase in the prevalence of pelvic floor dysfunction in females with sarcopenia, and suggested that the severity of pelvic floor dysfunction may be related to sarcopenia [27]. This study also found a correlation between incontinence and sarcopenia in elderly people, but the diagnostic parameters were different. Calf circumference gradually reduces with aging and muscle loss, when it reduces to the first critical value (< 34 cm for males, < 33 cm for females), the population may suffer from sarcopenia. At this time the pelvic floor muscles may not be seriously weakened and no incontinence symptoms will occur. However, if sarcopenia was not treated effectively and the calf circumference continued to shrink to the second critical value (< 28.5 cm for males, < 26.5 cm for females), the pelvic floor muscles would become sufficiently weakened to incur incontinence symptoms. In this study, the proportion of males with calf circumference < 28.5 cm was 18.5%, and females with calf circumference < 26.5 cm was 38.0%, indicating that muscular dystrophy in the elderly population was relatively serious, and should be met with intervention and timely treatment. At the same time, it also indicated that the diagnostic parameters of calf circumference < 28.5 cm for males and < 26.5 cm for females proposed in this study were reliable and could be used as a screening index of incontinence in the elderly population. We suggest carrying out large-scale screening of calf circumference during physical examination of the elderly population, detecting risk of incontinence, and providing prevention guidance early, to improve the quality of life for elderly.

The study found that the risk of incontinence was significantly reduced in normal BMI and overweight and obese individuals compared with those who were thinner. Current research had been inconsistent on the relationship between BMI and incontinence. The survey results of adult Chinese females by Peking Union Medical College Hospital showed that overweight and obese females were more likely to develop stress UI than those with normal BMI, and BMI $\ge$ 24 kg/m^2^ was a risk factor for FI [13, 30]. However, a recent study on female UI and pelvic floor muscle strength found that pelvic floor muscle strength would increase with the increase of BMI [31]. This contradiction may be caused by the different study populations. The elderly population, who often suffer multiple diseases, is different from the adult population, so the study of a single disease could not be conducted in isolation. Sarcopenia has been demonstrated to be prevalent in older females with UI and may be a clinical predictor of incontinence, influencing the treatment and management of the disease [32]. In this study, the relationship between BMI and incontinence cannot be generalized, and overweight/obesity may be a risk factor for incontinence in normal adult populations. For the elderly population with sarcopenia, overweight/obesity may be a protective factor for incontinence, which needs to be further discussed after the study population and indicators are refined. Therefore, we suggest that screening and intervention for sarcopenia should be carried out at the same time as the diagnosis of incontinence in elderly people [27]. Only the treatment of both diseases can achieve better results; otherwise, the presence of sarcopenia may affect the treatment effect of incontinence. The elderly people should improve their bad living habits and give up smoking and drinking. Varieties of exercises can be engaged to improve physical function, including resistance exercise, aerobic exercise, Kegel exercise, passive exercise, etc. Balanced diet and increasing the intake of high-quality protein, amino acids, vitamin D and other nutrients are suggested. Exercise combined with nutritional intervention can prevent and treat the sarcopenia, so as to reduce the incidence of incontinence caused by sarcopenia [33].

The limitation of this study is that the data come from the national general survey of the health status of the elderly population, and not a specially designed study for incontinence and sarcopenia. The incontinence-related data are incomplete and data from the International Consultation on Incontinence Questionnaire (ICIQ) is lacking. Moreover, it is a cross-sectional study. To clarify the relationship between sarcopenia and incontinence in elderly people, large-scale prospective cohort studies with different populations should be designed to obtain conclusions with a higher level of evidence.

## Conclusions

Incontinence in the elderly population is closely related to sarcopenia, and calf circumference is a convenient screening index of incontinence among elderly people. Screening and treatment of sarcopenia are routinely performed in elderly patients with incontinence. This study for the first time proposes that calf circumference < 28.5 cm in males and < 26.5 cm in females is a risk factor for incontinence in the elderly population, and it is suggested to measure calf circumference in routine physical examination of the elderly population and timely interventions should be made to reduce the risk of incontinence in subjects with calf circumference less than the threshold.

## Supplementary Information

**Additional file 1: Supplementary Table 1.** Coordinate points of ROC curve and Youden index in male population using logistic regression model. **Supplementary Table 2.** Coordinate points of ROC curve and Youden index in female population using logistic regression model. **Supplementary Table 3.** Association of calf circumference (continuous variable) and incontinence by sex. **Supplementary Table 4.** Association of calf circumference (cut-off points: 34 cm for male and 33 cm for female) and incontinence by sex. **Supplementary Table 5.** Association of calf circumference (cut-off points: 28.5 cm for male and 26.5 cm for female) and incontinence by sex.

## Data Availability

The data underlying this article will be shared on reasonable request to the corresponding author.
