# Supplementary material for: Association between calf circumference and incontinence in Chinese elderly
Source: BMC Public Health. 2023 Mar 11;23:471. doi: 10.1186/s12889-023-15324-4 (PMC10007784; doi:10.1186/s12889-023-15324-4)
Supplement: Supplementary file 1 — Additional file 1: Supplementary Table 1. Coordinate points of ROC curve and Youden index in male population using logistic regression model. Supplementary Table 2. Coordinate points of ROC curve and Youden index in female population using logistic regression model. Supplementary Table 3. Association of calf circumference (continuous variable) and incontinence by sex. Supplementary Table 4. Association of calf circumference (cut-off points: 34 cm for male and 33 cm for female) and incontinence by sex. Supplementary Table 5. Association of calf circumference (cut-off points: 28.5 cm for male and 26.5 cm for female) and incontinence by sex. [file 12889_2023_15324_MOESM1_ESM.docx]

| **Supplementary Table 1.**  Coordinate points of ROC curve and Youden index in male population using logistic regression model. | | | |
| --- | --- | --- | --- |
| Calf circumference | Sensitivity | 1 - Specificity | Youden Index |
| 0.00 | 1.000 | 1.000 | 0.000 |
| 1.50 | 1.000 | 0.997 | 0.003 |
| 2.50 | 1.000 | 0.997 | 0.002 |
| 5.00 | 0.999 | 0.997 | 0.002 |
| 8.00 | 0.999 | 0.997 | 0.002 |
| 9.50 | 0.999 | 0.997 | 0.002 |
| 10.50 | 0.999 | 0.997 | 0.001 |
| 11.50 | 0.998 | 0.997 | 0.001 |
| 12.50 | 0.996 | 0.991 | 0.005 |
| 13.50 | 0.995 | 0.988 | 0.007 |
| 14.50 | 0.994 | 0.988 | 0.006 |
| 15.50 | 0.993 | 0.988 | 0.005 |
| 16.50 | 0.993 | 0.982 | 0.010 |
| 17.50 | 0.992 | 0.982 | 0.010 |
| 18.50 | 0.989 | 0.974 | 0.016 |
| 19.50 | 0.987 | 0.974 | 0.014 |
| 20.50 | 0.977 | 0.938 | 0.039 |
| 21.50 | 0.972 | 0.927 | 0.046 |
| 22.50 | 0.967 | 0.891 | 0.075 |
| 23.50 | 0.957 | 0.845 | 0.113 |
| 24.50 | 0.944 | 0.815 | 0.128 |
| 25.50 | 0.925 | 0.783 | 0.142 |
| 26.50 | 0.900 | 0.730 | 0.170 |
| 27.50 | 0.870 | 0.677 | 0.192 |
| 28.50 | 0.823 | 0.604 | 0.219 |
| 29.50 | 0.772 | 0.557 | 0.215 |
| 30.50 | 0.662 | 0.455 | 0.207 |
| 31.50 | 0.592 | 0.393 | 0.199 |
| 32.50 | 0.498 | 0.320 | 0.178 |
| 33.50 | 0.421 | 0.264 | 0.157 |
| 34.50 | 0.349 | 0.214 | 0.135 |
| 35.50 | 0.266 | 0.141 | 0.125 |
| 36.50 | 0.199 | 0.117 | 0.082 |
| 37.50 | 0.160 | 0.103 | 0.057 |
| 38.50 | 0.115 | 0.085 | 0.030 |
| 39.50 | 0.091 | 0.076 | 0.015 |
| 40.50 | 0.055 | 0.050 | 0.005 |
| 41.50 | 0.046 | 0.047 | -0.001 |
| 42.50 | 0.036 | 0.044 | -0.008 |
| 43.50 | 0.030 | 0.038 | -0.008 |
| 44.50 | 0.026 | 0.035 | -0.009 |
| 45.50 | 0.019 | 0.021 | -0.002 |
| 46.50 | 0.016 | 0.021 | -0.005 |
| 47.50 | 0.015 | 0.021 | -0.006 |
| 48.50 | 0.011 | 0.018 | -0.006 |
| 49.50 | 0.010 | 0.018 | -0.008 |
| 50.50 | 0.007 | 0.012 | -0.005 |
| 51.50 | 0.006 | 0.006 | 0.000 |
| 52.50 | 0.005 | 0.006 | -0.001 |
| 53.50 | 0.004 | 0.006 | -0.002 |
| 54.50 | 0.004 | 0.003 | 0.001 |
| 55.50 | 0.003 | 0.003 | 0.000 |
| 57.00 | 0.003 | 0.003 | 0.000 |
| 58.50 | 0.003 | 0.003 | 0.000 |
| 59.50 | 0.002 | 0.003 | -0.001 |
| 61.00 | 0.002 | 0.003 | -0.001 |
| 63.00 | 0.002 | 0.003 | -0.001 |
| 65.50 | 0.002 | 0.000 | 0.002 |
| 67.50 | 0.002 | 0.000 | 0.002 |
| 69.00 | 0.001 | 0.000 | 0.001 |
| 73.00 | 0.001 | 0.000 | 0.001 |
| 77.00 | 0.001 | 0.000 | 0.001 |
| 79.00 | 0.001 | 0.000 | 0.001 |
| 80.50 | 0.000 | 0.000 | 0.000 |
| 86.50 | 0.000 | 0.000 | 0.000 |
| 93.00 | 0.000 | 0.000 | 0.000 |

| **Supplementary Table 2.**  Coordinate points of ROC curve and Youden index in female population using logistic regression model. | | | |
| --- | --- | --- | --- |
| Calf circumference | Sensitivity | 1 - Specificity | Youden Index |
| 1.00 | 1.000 | 1.000 | 0.000 |
| 2.50 | 1.000 | 0.999 | 0.001 |
| 4.50 | 0.999 | 0.999 | 0.001 |
| 6.50 | 0.999 | 0.999 | 0.000 |
| 7.50 | 0.998 | 0.996 | 0.003 |
| 8.50 | 0.998 | 0.996 | 0.002 |
| 9.50 | 0.998 | 0.993 | 0.005 |
| 10.50 | 0.997 | 0.991 | 0.006 |
| 11.50 | 0.996 | 0.991 | 0.005 |
| 12.50 | 0.995 | 0.990 | 0.005 |
| 13.50 | 0.994 | 0.987 | 0.007 |
| 14.50 | 0.993 | 0.986 | 0.007 |
| 15.50 | 0.991 | 0.976 | 0.015 |
| 16.50 | 0.988 | 0.969 | 0.019 |
| 17.50 | 0.984 | 0.953 | 0.031 |
| 18.50 | 0.976 | 0.935 | 0.041 |
| 19.50 | 0.972 | 0.922 | 0.050 |
| 20.50 | 0.954 | 0.852 | 0.101 |
| 21.50 | 0.940 | 0.817 | 0.123 |
| 22.50 | 0.919 | 0.747 | 0.171 |
| 23.50 | 0.893 | 0.682 | 0.211 |
| 24.50 | 0.854 | 0.615 | 0.239 |
| 25.50 | 0.801 | 0.536 | 0.266 |
| 26.50 | 0.741 | 0.455 | 0.286 |
| 27.50 | 0.679 | 0.401 | 0.279 |
| 28.50 | 0.595 | 0.335 | 0.260 |
| 29.50 | 0.534 | 0.293 | 0.241 |
| 30.50 | 0.413 | 0.205 | 0.209 |
| 31.50 | 0.355 | 0.172 | 0.183 |
| 32.50 | 0.283 | 0.126 | 0.156 |
| 33.50 | 0.225 | 0.109 | 0.116 |
| 34.50 | 0.176 | 0.104 | 0.072 |
| 35.50 | 0.129 | 0.072 | 0.057 |
| 36.50 | 0.096 | 0.063 | 0.033 |
| 37.50 | 0.077 | 0.055 | 0.022 |
| 38.50 | 0.056 | 0.044 | 0.012 |
| 39.50 | 0.047 | 0.041 | 0.006 |
| 40.50 | 0.031 | 0.023 | 0.008 |
| 41.50 | 0.026 | 0.021 | 0.005 |
| 42.50 | 0.020 | 0.018 | 0.002 |
| 43.50 | 0.018 | 0.017 | 0.001 |
| 44.50 | 0.016 | 0.016 | 0.001 |
| 45.50 | 0.013 | 0.013 | 0.000 |
| 46.50 | 0.012 | 0.013 | -0.001 |
| 47.50 | 0.012 | 0.013 | -0.001 |
| 48.50 | 0.009 | 0.011 | -0.002 |
| 49.50 | 0.009 | 0.011 | -0.002 |
| 50.50 | 0.006 | 0.004 | 0.002 |
| 51.50 | 0.005 | 0.004 | 0.001 |
| 52.50 | 0.004 | 0.004 | 0.000 |
| 54.00 | 0.004 | 0.004 | 0.000 |
| 55.50 | 0.004 | 0.003 | 0.001 |
| 57.50 | 0.003 | 0.003 | 0.001 |
| 59.50 | 0.003 | 0.003 | 0.001 |
| 60.50 | 0.003 | 0.003 | 0.000 |
| 62.50 | 0.002 | 0.003 | 0.000 |
| 64.50 | 0.002 | 0.003 | -0.001 |
| 67.50 | 0.002 | 0.003 | -0.001 |
| 71.00 | 0.002 | 0.001 | 0.000 |
| 73.00 | 0.001 | 0.001 | 0.000 |
| 75.50 | 0.001 | 0.001 | 0.000 |
| 77.50 | 0.001 | 0.001 | 0.000 |
| 78.50 | 0.001 | 0.001 | -0.001 |
| 79.50 | 0.000 | 0.001 | -0.001 |
| 80.50 | 0.000 | 0.001 | -0.001 |
| 83.00 | 0.000 | 0.001 | -0.001 |

| **Supplementary Table 3.** Association of calf circumference (continuous variable) and incontinence by sex | | | | | | | | | |
| --- | --- | --- | --- | --- | --- | --- | --- | --- | --- |
| Variable | Male | | | |  | Female | | | |
|  | Beta | OR | 95%CI | |  | Beta | OR | 95%CI | |
|  |  |  | Lower | Upper |  |  |  | Lower | Upper |
| **Model 1** |  |  |  |  |  |  |  |  |  |
| Calf circumference^1^ | -0.0761 | 0.927 | 0.910 | 0.944 |  | -0.0949 | 0.909 | 0.897 | 0.922 |
| **Model 2** |  |  |  |  |  |  |  |  |  |
| Calf circumference^1^ | -0.0289 | 0.972 | 0.948 | 0.996 |  | -0.0146 | 0.985 | 0.967 | 1.004 |
| Age group (70-80) | -0.4670 | 2.437 | 0.895 | 8.605 |  | -0.9237 | 2.720 | 0.746 | 17.522 |
| Age group (80-90) | -0.1134 | 3.470 | 1.337 | 11.937 |  | 0.3290 | 9.518 | 2.894 | 58.914 |
| Age group (90-100) | 0.6474 | 7.427 | 2.919 | 25.280 |  | 0.9771 | 18.197 | 5.602 | 112.070 |
| Age group (>100) | 1.2908 | 14.133 | 5.461 | 48.613 |  | 1.5417 | 32.005 | 9.848 | 197.188 |
| Have a history of falling | 0.1499 | 1.350 | 1.013 | 1.789 |  | -0.00618 | 0.988 | 0.795 | 1.223 |
| Have a history of smoking | -0.0780 | 0.856 | 0.640 | 1.143 |  | 0.0208 | 1.043 | 0.731 | 1.463 |
| Have a history of drinking | -0.0123 | 0.976 | 0.722 | 1.314 |  | -0.0115 | 0.977 | 0.708 | 1.333 |
| Regularly Exercise | -0.1901 | 0.684 | 0.507 | 0.917 |  | -0.1277 | 0.775 | 0.601 | 0.992 |
| Daily activity limited | 0.8515 | 5.490 | 3.968 | 7.724 |  | 0.9601 | 6.823 | 5.123 | 9.249 |
| Socioeconomic Status (Average) | -0.2853 | 0.778 | 0.533 | 1.154 |  | -0.0112 | 0.598 | 0.459 | 0.784 |
| Socioeconomic Status (Better) | 0.0170 | 0.575 | 0.355 | 0.933 |  | -0.4921 | 0.370 | 0.249 | 0.543 |
| Waist Circumference (>=80/85) | 0.1889 | 1.459 | 1.081 | 1.973 |  | 0.0286 | 1.059 | 0.854 | 1.315 |
| Frequently have Vegetable & Fruit | -0.2459 | 0.611 | 0.359 | 1.075 |  | -0.2890 | 0.561 | 0.388 | 0.820 |
| Education (Primary or junior high school) | -0.1519 | 0.830 | 0.618 | 1.116 |  | -0.0533 | 1.390 | 1.035 | 1.854 |
| Education (University or above) | 0.1180 | 1.088 | 0.689 | 1.693 |  | 0.4363 | 2.269 | 1.332 | 3.759 |
| BMI level (Normal) | 0.0329 | 0.722 | 0.438 | 1.161 |  | -0.3798 | 0.539 | 0.425 | 0.682 |
| BMI level (Overweight or obesity) | -0.3920 | 0.472 | 0.344 | 0.644 |  | 0.1422 | 0.909 | 0.629 | 1.295 |
| Have a history of Respiratory disease | 0.1588 | 1.374 | 0.960 | 1.940 |  | 0.0823 | 1.179 | 0.836 | 1.639 |
| Have a history of Urinary system disease | 0.2170 | 1.543 | 1.045 | 2.244 |  | -0.5482 | 0.334 | 0.052 | 1.176 |
| Have a history of Stroke or cardiovascular disease | 0.4965 | 2.700 | 1.946 | 3.728 |  | 0.5145 | 2.798 | 2.096 | 3.722 |
| Have a history of Nerves system disease | 0.7779 | 4.739 | 2.962 | 7.525 |  | 0.7580 | 4.554 | 3.295 | 6.292 |
| Have a history of Pregnant | / | / | / | / |  | 0.0737 | 1.159 | 0.585 | 2.512 |
| Note: Calf circumference (cm)^1^: The calf circumference was included in the model as a continuous variable. Model 1 was univariate model; Model 2 further adjusted age, history of falling, smoking status, drinking status, physical exercise, limited ability of daily activities, intake of vegetables and fruits, socioeconomic status, waistline circumference, BMI, education, history of respiratory diseases, history of urinary system diseases, history of stroke or cardiovascular diseases, history of nerves system disease and history of pregnant (only in females). | | | | | | | | | |

| **Supplementary Table 4.** Association of calf circumference (cut-off points: 34 cm for male and 33 cm for female) and incontinence by sex | | | | | | | | | |
| --- | --- | --- | --- | --- | --- | --- | --- | --- | --- |
| Variable | Male | | | |  | Female | | | |
|  | Beta | OR | 95%CI | |  | Beta | OR | 95%CI | |
|  |  |  | Lower | Upper |  |  |  | Lower | Upper |
| **Model 1** |  |  |  |  |  |  |  |  |  |
| Calf circumference (< 34/33 cm) | 0.3537 | 2.029 | 1.593 | 2.607 |  | 0.5007 | 2.722 | 2.181 | 3.440 |
| **Model 2** |  |  |  |  |  |  |  |  |  |
| Calf circumference (< 34/33 cm) | 0.0985 | 1.218 | 0.882 | 1.691 |  | 0.0556 | 1.118 | 0.820 | 1.540 |
| Age group (70-80) | -0.4732 | 2.458 | 0.904 | 8.670 |  | -0.9290 | 2.733 | 0.749 | 17.616 |
| Age group (80-90) | -0.1107 | 3.532 | 1.363 | 12.136 |  | 0.3239 | 9.568 | 2.906 | 59.250 |
| Age group (90-100) | 0.6503 | 7.561 | 2.974 | 25.718 |  | 0.9814 | 18.466 | 5.676 | 113.818 |
| Age group (>100) | 1.3064 | 14.572 | 5.640 | 50.063 |  | 1.5582 | 32.875 | 10.103 | 202.675 |
| Have a history of falling | 0.1483 | 1.345 | 1.010 | 1.782 |  | -0.00515 | 0.990 | 0.797 | 1.226 |
| Have a history of smoking | -0.0723 | 0.865 | 0.647 | 1.156 |  | 0.0220 | 1.045 | 0.733 | 1.466 |
| Have a history of drinking | -0.0111 | 0.978 | 0.724 | 1.317 |  | -0.0105 | 0.979 | 0.709 | 1.335 |
| Regularly Exercise | -0.1902 | 0.684 | 0.506 | 0.917 |  | -0.1281 | 0.774 | 0.600 | 0.992 |
| Daily activity limited | 0.8565 | 5.546 | 4.009 | 7.800 |  | 0.9647 | 6.886 | 5.172 | 9.331 |
| Socioeconomic Status (Average) | 0.0162 | 0.772 | 0.529 | 1.145 |  | -0.0134 | 0.593 | 0.455 | 0.777 |
| Socioeconomic Status (Better) | -0.2911 | 0.568 | 0.350 | 0.921 |  | -0.4952 | 0.366 | 0.247 | 0.539 |
| Waist Circumference (>=80/85) | 0.1737 | 1.415 | 1.050 | 1.911 |  | 0.0133 | 1.027 | 0.832 | 1.269 |
| Frequently have Vegetable & Fruit | -0.2548 | 0.601 | 0.352 | 1.056 |  | -0.2968 | 0.552 | 0.383 | 0.806 |
| Education (Primary or junior high school) | -0.1588 | 0.815 | 0.608 | 1.094 |  | -0.0599 | 1.379 | 1.026 | 1.838 |
| Education (University or above) | 0.1135 | 0.689 | 0.418 | 1.111 |  | 0.4411 | 2.276 | 1.336 | 3.770 |
| BMI level (Normal) | -0.3984 | 0.457 | 0.334 | 0.623 |  | -0.3868 | 0.529 | 0.417 | 0.667 |
| BMI level (Overweight or obesity) | 0.0133 | 0.689 | 0.418 | 1.111 |  | 0.1366 | 0.893 | 0.617 | 1.273 |
| Have a history of Respiratory disease | 0.1538 | 1.360 | 0.951 | 1.919 |  | 0.0788 | 1.171 | 0.831 | 1.627 |
| Have a history of Urinary system disease | 0.2235 | 1.564 | 1.060 | 2.272 |  | -0.5574 | 0.328 | 0.051 | 1.158 |
| Have a history of Stroke or cardiovascular disease | 0.4911 | 2.670 | 1.927 | 3.684 |  | 0.5120 | 2.784 | 2.086 | 3.703 |
| Have a history of Nerves system disease | 0.7814 | 4.773 | 2.988 | 7.567 |  | 0.7614 | 4.585 | 3.319 | 6.333 |
| Have a history of Pregnant | / | / | / | / |  | 0.0740 | 1.160 | 0.585 | 2.512 |
| Note: Calf circumference (cm)^1^: The calf circumference was included in the model as a binomial variable. Model 1 was univariate model; Model 2 further adjusted age, history of falling, smoking status, drinking status, physical exercise, limited ability of daily activities, intake of vegetables and fruits, socioeconomic status, waistline circumference, BMI, education, history of respiratory diseases, history of urinary system diseases, history of stroke or cardiovascular diseases, history of nerves system disease and history of pregnant (only in females). | | | | | | | | | |

| **Supplementary Table 5.** Association of calf circumference (cut-off points: 28.5 cm for male and 26.5 cm for female) and incontinence by sex | | | | | | | | | |
| --- | --- | --- | --- | --- | --- | --- | --- | --- | --- |
| Variable | Male | | | |  | Female | | | |
|  | Beta | OR | 95%CI | |  | Beta | OR | 95%CI | |
|  |  |  | Lower | Upper |  |  |  | Lower | Upper |
| **Model 1** |  |  |  |  |  |  |  |  |  |
| Calf circumference (< 34/33 cm) | 0.5560 | 3.040 | 2.420 | 3.809 |  | 0.6159 | 3.427 | 2.930 | 4.010 |
| **Model 2** |  |  |  |  |  |  |  |  |  |
| Calf circumference (< 34/33 cm) | 0.2414 | 1.620 | 1.197 | 2.188 |  | 0.1370 | 1.315 | 1.056 | 1.637 |
| Age group (70-80) | -0.4606 | 2.430 | 0.892 | 8.582 |  | -0.9150 | 2.755 | 0.755 | 17.755 |
| Age group (80-90) | -0.1123 | 3.443 | 1.325 | 11.846 |  | 0.3347 | 9.613 | 2.921 | 59.517 |
| Age group (90-100) | 0.6448 | 7.340 | 2.883 | 24.993 |  | 0.9784 | 18.298 | 5.630 | 112.711 |
| Age group (>100) | 1.2766 | 13.807 | 5.331 | 47.512 |  | 1.5303 | 31.775 | 9.775 | 195.790 |
| Have a history of falling | 0.1506 | 1.351 | 1.014 | 1.791 |  | -0.00446 | 0.991 | 0.798 | 1.228 |
| Have a history of smoking | -0.0777 | 0.856 | 0.640 | 1.144 |  | 0.0192 | 1.039 | 0.728 | 1.459 |
| Have a history of drinking | -0.00915 | 0.982 | 0.727 | 1.323 |  | -0.00897 | 0.982 | 0.711 | 1.339 |
| Regularly Exercise | -0.1836 | 0.693 | 0.513 | 0.929 |  | -0.1227 | 0.782 | 0.606 | 1.003 |
| Daily activity limited | 0.8473 | 5.444 | 3.933 | 7.661 |  | 0.9558 | 6.764 | 5.078 | 9.171 |
| Socioeconomic Status (Average) | 0.0112 | 0.775 | 0.531 | 1.150 |  | -0.0138 | 0.595 | 0.457 | 0.780 |
| Socioeconomic Status (Better) | -0.2777 | 0.580 | 0.358 | 0.942 |  | -0.4911 | 0.369 | 0.249 | 0.543 |
| Waist Circumference (>=80/85) | 0.2021 | 1.498 | 1.109 | 2.028 |  | 0.0384 | 1.080 | 0.871 | 1.340 |
| Frequently have Vegetable & Fruit | -0.2463 | 0.611 | 0.359 | 1.071 |  | -0.2886 | 0.561 | 0.388 | 0.821 |
| Education (Primary or junior high school) | -0.1566 | 0.811 | 0.604 | 1.090 |  | -0.0486 | 1.401 | 1.042 | 1.868 |
| Education (University or above) | 0.1041 | 1.053 | 0.668 | 1.637 |  | 0.4344 | 2.271 | 1.334 | 3.759 |
| BMI level (Normal) | -0.3691 | 0.487 | 0.355 | 0.666 |  | -0.3614 | 0.553 | 0.436 | 0.700 |
| BMI level (Overweight or obesity) | 0.0184 | 0.717 | 0.437 | 1.149 |  | 0.1311 | 0.906 | 0.627 | 1.288 |
| Have a history of Respiratory disease | 0.1572 | 1.369 | 0.956 | 1.936 |  | 0.0888 | 1.194 | 0.847 | 1.661 |
| Have a history of Urinary system disease | 0.2283 | 1.579 | 1.069 | 2.297 |  | -0.5436 | 0.337 | 0.053 | 1.182 |
| Have a history of Stroke or cardiovascular disease | 0.4934 | 2.683 | 1.934 | 3.705 |  | 0.5139 | 2.795 | 2.092 | 3.718 |
| Have a history of Nerves system disease | 0.7816 | 4.774 | 2.979 | 7.592 |  | 0.7572 | 4.547 | 3.288 | 6.286 |
| Have a history of Pregnant | / | / | / | / |  | 0.0743 | 1.160 | 0.586 | 2.511 |
| Note: Calf circumference (cm)^1^: The calf circumference was included in the model as a binomial variable. Model 1 was univariate model; Model 2 further adjusted age, history of falling, smoking status, drinking status, physical exercise, limited ability of daily activities, intake of vegetables and fruits, socioeconomic status, waistline circumference, BMI, education, history of respiratory diseases, history of urinary system diseases, history of stroke or cardiovascular diseases, history of nerves system disease and history of pregnant (only in females). | | | | | | | | | |
